# Diabetic Ketoacidosis as the Presentation of Immune Dysregulation, Polyendocrinopathy, Enteropathy, and X-Linked Syndrome: A Case Report

**DOI:** 10.1155/crpe/8888683

**Published:** 2025-10-03

**Authors:** Nicola Wolff

**Affiliations:** Paediatric A&E, Royal London Hospital, London, UK

## Abstract

Neonatal diabetes mellitus (NDM) is a rare and often misdiagnosed collection of disorders typically presenting in the first 6 months of life, characterized by insulin deficiency and hyperglycemia. This case study discusses a neonate initially suspected of having sepsis due to nonspecific symptoms such as vomiting, increased work of breathing, and grunting. Upon further investigation, the child was diagnosed with neonatal diabetic ketoacidosis (DKA) and treated accordingly. Genetic testing and subsequent management revealed that the infant was hemizygous for a *FOXP3* missense variant, leading to a diagnosis of IPEX syndrome, an X-linked autoimmune disorder with a poor prognosis. This case highlights the importance of considering NDM in differential diagnoses, especially when symptoms mimic other neonatal illnesses such as sepsis, and emphasizes the need for prompt recognition and management to prevent complications. In addition, this article outlines the management strategies for IPEX syndrome, including insulin therapy, immune suppression, and the potential for hematopoietic stem cell transplantation.

## 1. Background

Neonatal diabetes mellitus (NDM) is a rare collection of disorders that are most often diagnosed in the first 6 months of life [1]. It is characterized by low levels of insulin in the blood, leading to hyperglycemia, with an incidence of 1 in 90,000 live births [2]. Two clinical presentations have been described: a transient form and a permanent form. This disease is caused by two major mechanisms: malformation of the pancreas, or abnormal function of pancreatic beta cells and thus poor insulin production [2]. 50%–60% of neonatal diabetes cases are transient and recover within a year [3, 4]. The presentation of NDM can mimic sepsis and other causes of neonatal collapse, which can make the diagnosis difficult [3]. It is important to keep an open mind during the resuscitation of the unwell neonate. I will discuss a case in which a neonate presented with nonspecific symptoms and was initially believed to be septic.

## 2. Case Study

An 8-day-old baby presented to the accident and emergency with vomiting and increased work of breathing, following a choking episode the previous evening. While breastfeeding, he had a brief episode of choking. There was no color change, apnea, or loss of tone. He had been tachypneic since and was reviewed by the community midwife, who was concerned about the work of breathing and advised attendance at A&E. He was having nonprojectile yellow–green vomit after every feed and had a 12% weight loss (birth weight 2.90 kg; weight at presentation 2.55 kg). He was having normal bowel habit and urine output. In the department, the baby was intermittently grunting.

The baby was born at 40 weeks of gestation via a Category 1 caesarean section, performed due to fetal bradycardia. Despite this, he was born in good condition, and no resuscitation was required. During pregnancy, an ultrasound showed hyperechogenic bowel and short long bones; however, his parents declined amniocentesis. The parents were nonconsanguineous, and his 2-year-old brother was fit and healthy.

While in A&E, the patient began continuously grunting with a respiratory rate of > 60 and a heart rate of 180, though his oxygen saturations remained > 94% in room air. Increased work of breathing was noted, but the chest was clear on auscultation. He had signs of severe dehydration with a capillary refill time (CRT) of 3–4 s centrally, a sunken fontanelle, and dry mucous membranes. The Moro reflex could not be elicited. Heart sounds were normal, and abdominal examination was unremarkable.

The patient was treated for suspected sepsis, a cannula was sited, and a fluid bolus and antibiotics were started. Blood samples were sent, and the initial venous blood gas showed a severe metabolic acidosis:

pH 6.937 pCO2 2.05 kPa pO2 9 kPa BE -31.3 mmol/L HCO3 3.1 mmol/L ketones 3.4 mmol/L glucose 50 mmol/L Na 146 mmol/l K+ 7.3 mmol/L Hb 166 g/L.

The patient was moved to Resus, and a medical emergency call was placed. He was treated for neonatal diabetic ketoacidosis (DKA). He received two 10 mLs/kg fluid boluses followed by fluids as per BSPED guidelines. Salbutamol nebulizers were given to treat the hyperkalemia. The continued grunting was managed with the application of continuous positive airway pressure (CPAP). The patient's temperature was initially unrecordable, and he was warmed under the Resuscitaire. There was a discussion around whether calcium gluconate should be started as management for the high potassium. However, it was felt not necessary, given the high ionized calcium on the VBG and the expectation of its resolution associated with the insulin infusion. One hour after the fluids were commenced, insulin infusion was started at 0.05 units/kg/hour. An arterial line was placed to facilitate repeated blood gases, due to the difficulty of repeatedly taking blood from a small baby. The patient was admitted to the PICU from A&E, and management for DKA was continued. A second line was placed due to the likelihood of ongoing insulin requirement. He was started on an insulin pump 12 days after admission with diluted insulin 1:10 ratio, basal rate 0.35 units per hour, and a glucose target of 5.6 mmol/L. The ICR ratio was 1:3.2, and the ISF was 1:4.5.

An abdominal ultrasound was performed and was unremarkable. The pancreas could not be fully visualized, but normal pancreatic tissue was seen in the region of the pancreatic head. The initial genetic testing for common mutations causing neonatal diabetes (*6q24, ABCC8, INS,* and *KCNJ11*) was normal. The testing for the 6q24 transient neonatal diabetes critical region revealed normal allelic methylation and normal dosage. The patient was discharged from the hospital following successful glucose control and diabetic education of both parents.

At the age of 7 weeks, he had further choking episodes during feeding and was seen twice by his GP, but given no improvement, it was recommended that he be seen in A&E. His feeding had reduced from 100–120 mLs consumed in 30 min every 3–4 h to 70 mLs consumed in 2 h. There was no coughing in between feeding, and he had continued to wake for feeds. There was no color change, no vomiting, no sweating during feeding, and no fever but some loose stools. Examination and VBG were grossly normal with a very mild work of breathing. In the 2 weeks prior to admission, his weight fell from the 9th centile to 0.4th centile, a loss of 150 g. Chest X-ray showed normal cardiothymic contour with patchy changes bi-basally and in the right upper lobe.

A formal assessment by Speech and Language Therapy determined his swallow to be unsafe, and so he was started on NG feeding. Tube oesophagram was normal, with no evidence of a tracheoesophageal fistula. Genetics found that he was hemizygous for the *FOXP3* missense variant *P.(Thr380lle)*, and he was diagnosed with immune dysregulation, polyendocrinopathy, enteropathy, and X-linked syndrome (IPEX). His father was investigated and found to be a carrier, and his 2-year-old brother was tested and was genetically normal.

### 2.1. IPEX Syndrome

IPEX is a genetic disorder characterized by early onset systemic autoimmunity [5]. It is caused by a hemizygous pathogenic variant in *FOXP3*, the gene which codes for the P3 transcription factor, which is located on the X chromosome [5]. This transcription factor is vital in the development and maintenance of CD4-positive regulatory T cells. Uncontrolled T cell activation leads to autoimmunity and inflammation [6]. This affects many systems, including severe gastrointestinal disease, endocrinopathies, and dermatitis [5]. Unlike in the case presented here, watery diarrhea and malabsorption are the most common initial symptoms of IPEX, due to loss of intestinal villi structure [7]. There has been a documented case of a patient with IPEX syndrome presenting with DKA at 3 weeks of age [8]. I have not encountered a publication of a patient with IPEX syndrome presenting with DKA earlier than 3 weeks, as in this case. It is unclear whether the early presentation of DKA indicates a poorer prognosis than other patients with IPEX syndrome; though it has been found that those with disease onset after the first year usually experience less severe manifestations [9].

The prognosis of IPEX syndrome is poor, with many patients dying in the first or second years of life [10]. Supportive care is vital in the management of IPEX syndrome, as it affects many of the body's systems. Total parenteral nutrition (TPN) support is required until immune suppression is able to reestablish intestinal function [7]. The treatment of Type 1 diabetes mellitus and autoimmune thyroid disease are as standard, with insulin and thyroxin [7]. Skin conditions are managed with topical therapies, such as steroids and emollients [7]. An early hematopoetic stem cell transplant can potentially be curative, and there is ongoing research into gene therapy for IPEX syndrome [10].

## 3. Conclusion

This case highlights the diagnostic challenges and clinical complexity of NDM, particularly when its presentation mimics more common neonatal conditions such as sepsis. It underscores the importance of maintaining a broad differential diagnosis during the resuscitation and management of neonates with nonspecific symptoms. Early recognition and treatment of neonatal DKA can significantly impact outcomes, as demonstrated by the successful management of the case. In addition, the unexpected diagnosis of IPEX syndrome, a rare genetic disorder, emphasizes the need for thorough genetic evaluation in neonates with unexplained symptoms. Ultimately, this article demonstrates the critical role of timely diagnosis, appropriate management, and parental education in optimizing the care of neonates with rare metabolic and genetic disorders.
